# Rectal cancer: Short-term reproducibility of intravoxel incoherent motion parameters in 3.0T magnetic resonance imaging

**DOI:** 10.1097/MD.0000000000006866

**Published:** 2017-05-12

**Authors:** Hongliang Sun, Yanyan Xu, Qiaoyu Xu, Kaining Shi, Wu Wang

**Affiliations:** a Department of Radiology, China-Japan Friendship Hospital, Chaoyang District; b Philips Healthcare, Beijing, China.

**Keywords:** diffusion-weighted imaging, intravoxel incoherent motion, magnetic resonance imaging, perfusion, rectal cancer

## Abstract

The aim of this study was to evaluate the short-term test-retest reproducibility of diffusion-weighted magnetic resonance imaging (DW-MRI) parameters of rectal cancer with 3.0T MRI.

Twenty-six patients with rectal cancer underwent MRI, including diffusion-weighted imaging with 8 b values. Apparent diffusion coefficient (ADC) and intravoxel incoherent motion (IVIM) parameters (D, pure diffusion; f, perfusion fraction; D∗, pseudodiffusion coefficient) were, respectively, calculated. The short-term test-retest reproducibility, the intra and interobserver variation of the IVIM parameters were assessed based on the repeatability coefficient and Bland-Altman limits of agreement.

There was no significant intra or interobserver difference observed in the parameters on the same DW-MRI scan. The corresponding repeatability coefficient of intra- and interobserver analysis for ADC, D, f, and D∗ was 5.4%, 11.1%, 55.4%, and 40.3%; 10.9%, 41.6%, 134.0%, and 177.6%, respectively. The test-retest repeatability coefficient for ADC, D, f, and D∗ was 19.1%, 24.5%, 126.3%, and 197.4%, respectively, greater than the intraobserver values.

ADC and D have better short-term test-retest reproducibility than f and D∗. Considering the poor test-retest reproducibility for f and D,∗ variance in these 2 parameters should be interpreted with caution in longitudinal studies on rectal cancer in which treatment response and recurrence are monitored.

## 1 Introduction

Diffusion-weighted magnetic resonance imaging (DW-MRI) has been reported to be a useful approach for the assessment of chemoradiation therapy in cancers, but there are some conflicting reports.^[1–4]^ Traditional diffusion-weighted imaging (DWI) measures the diffusion of water quantitatively through a Gauss-distribution model based on the assumption of free diffusion, and does not fully account for tissue behavior in vivo. The calculated apparent diffusion coefficient (ADC) is influenced by both water diffusion and microcirculation within the normal capillary network perfusion effects.^[5]^ There is growing trend in applying variable analytical techniques beyond simple monoexponential model to tease out the effects of microcapillary perfusion from DW-MRI data. According to the intravoxel incoherent motion (IVIM) theory proposed by Le Bihan et al,^[6]^ the diffusion effect obtained in traditional DWI is caused not only by water molecule diffusion but also by the capillary network. The weight of perfusion effect in the entire apparent diffusion decreases as the b value increases. Thus, the pure diffusion and perfusion effect could be separated through multiple b values, and the 3 parameters diffusion coefficient (*D*), perfusion fraction (*f*), and pseudodiffusion coefficient (*D∗*) could be derived from a biexponential model.^[6,7]^

Currently, there is growing interest in applying DW-MRI to chemotherapy research.^[3,8]^ Although many published studies have shown the potential value of DWI parameters for assessment of therapy response,^[2–4,9]^ few have questioned its measurement reproducibility when applied, especially for IVIM. The measurement reproducibility reflects biological variation, observer errors, and instrumental errors. Knowledge of the measurement reproducibility is pivotal to better understand the changes in IVIM parameters that can be definitely ascribed to disease characterization or response assessment, and for its potential value as a imaging biomarker.

Hence, the purpose of this study was to prospectively determine the repeatability of DW-MRI relative parameters measurements derived from short-term test-retest DW-MR data for rectal cancer.

## 2 Materials and Methods

Institutional review board approval was obtained for this prospective study. All participants provided their written informed consent for publication. The authors retained full control of all the data collected and information submitted for publication.

### 2.1 Patients

Between August 2013 and April 2014, 35 patients with biopsy-proven rectal cancer underwent pelvic MR examination (including 8 *b* values DW sequence). We excluded cases who had: previous rectal surgery (n = 1); preexamination chemoradiotherapy or unidentified herbal medicine therapy (n = 1) for the rectal lesion; heavy intestinal peristalsis artifacts (n = 2); tumor stage T2 or earlier stage on MRI (n = 3); mucinous adenocarcinoma (n = 2). In total, 26 patients (17 men and 9 women; mean age, 59.8 years; age range, 38–79 years) were finally enrolled. According to the distance between the inferior part of the tumor and the anal verge, the rectal cancers are divided into 3 groups: upper (>10 cm), middle (5–10 cm), and lower (<5 cm). Our study included a total of 16 upper-middle rectal cancers and 10 lower ones.

For relative motionless organ in pelvic cavity, the repeatability of prostate DW-MRI-derived parameters for the recruited male patients was also analyzed as reference. However, one male patient with upper rectal cancer was excluded owing to the limited coverage of prostate tissue. Thus, only 16 male patients were included in the final analysis of prostate.

### 2.2 MR examination

The patients were on a low-residue diet before the examination and were asked to fast on the day of the examination. An intramuscular injection of 10-mg anisodamine hydrochloride was given to each patient to prevent intestine peristalsis. The patients were asked to remain steady to minimize possible motion artifacts or deformation during the examination. They were not repositioned between 2 DWI scans.

Pelvis MR scanning was performed on a 3T whole-body scanner (Ingenia, Philips Medical Systems, Best, the Netherlands) with a gradient strength of 45 mT/m and a gradient switching rate of 200 mT/m/ms, using a 16-channel anterior torso dS coil and a 16-channel posterior table dS coil. 2D sagittal and coronal T2W Turbo spin echo (TSE) sequences were obtained using the following parameters: repetition time (TR), 3761 ms; echo time (TE), 110 ms; field of view (FOV), 24 × 24 cm; slice thickness, 3 mm with a 0.3-mm gap; acquisition matrix, 336 × 252; NSA, 1. 2D axial T2W TSE sequences were obtained perpendicular to the tumoral axis in the sagittal view^[10–11]^: TR, 3865 ms; TE, 100 ms; FOV, 14 × 14 cm; slice thickness, 3 mm with a 0.3-mm gap; acquisition matrix, 232 × 228.

Axial DWI sequence perpendicular to the tumoral axis in the sagittal view was performed twice with parallel acquisiton technique (sensitivity encoding, SENSE), using a single-shot echo-planar imaging pulse sequence, with free breathing using the following parameters: TE/TR, 76/6000 ms; FOV, 20 × 30 cm; slice thickness, 4 mm with a 0.2-mm gap; acquisition matrix, 80 × 144; pixel size, 1.5 × 1.5 mm; NSA 2, eight b values (0, 25, 50, 75, 150, 400, 800, 1000 s/mm^2^). Frequency selection plus inversion recovery fat-suppression technique (spectral attenuated inversion recovery) was adopted in the DWI sequence. The scan time for a single DWI sequence was 6.3 minutes. The interval between 2 DWI scans was 20 to 30 minutes, and the relevant conventional MR scanning sequences mentioned before were completed during this time to reduce the total scan time.

### 2.3 Image processing and analysis

Conventional scan sequences were used for radiologic diagnosis and morphological evaluation, such as the depth of invasion, lymph node involvement, and treatment strategies selected.

The raw data from diffusion-weighted images were transferred to an EWS4.1 workstation and analyzed using the in-house software (IDL 6.3 software, Boulder, CO). The ADCs were obtained by using all b values (0–1000 s/mm^2^) fitted to monoexponential model, whereas IVIM parameters were calculated by a biexponential model described by Le Bihan et al^[6]^: 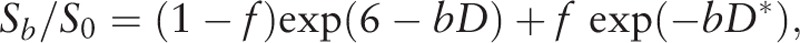


where *S*_*b*_ is the signal intensity in the pixel with diffusion gradient, *S*_*0*_ is the signal intensity in the pixel without diffusion gradient, *D* is the true diffusion as reflected by pure molecular diffusion, *f* is the perfusion fraction related to microcirculation, and *D∗* is the pseudodiffusion coefficient related to perfusion.

**Figure 1:**
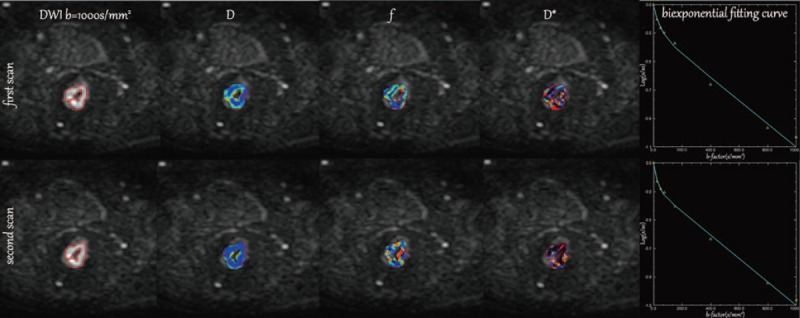
Twice-scanning images (including diffusion-weighted image [b = 1000 s/mm^2^], parametric IVIM color maps of rectal cancer [*D, f*, and *D∗*], plots of signal decay and biexponential fitting curves) of a 52-year-old male patient with middle-rectal cancer. Region of interests were manually drawn to contour the border of the rectal cancers on the slice (DWI images) with the maximum lesion size in twice-scanning. The upper line: the first examination images. The lower line: the second examination images.

Region of interest (ROIs) were manually drawn to contour the border of the rectal cancers on the slice (DWI images) with the maximum lesion size, avoiding the inclusion of intestinal gas and liquid, for 2 sequences by 2 independently experienced radiologists (10 years and 8 years in gastrointestinal imaging), respectively. Macroscopic necrosis, if any, was excluded. Meanwhile, another circular ROI (100 mm^2^) was drawn and placed free hand within the left gluteal muscle on the same slice selected above for the first DWI sequence. The DW-MRI-derived parameters’ values were calculated using the pixel-by-pixel fitting method and expressed as the mean values of all the pixels within the ROI (Fig. 1).

To assess intraobserver variability, the results of the first DWI sequence for all patients were delineated twice with an interval of at least 2 months between the delineations. Additionally, the DW-MRI-derived parameters of the largest slice for the prostate were simultaneously derived and recorded as reference values from 2 identical DWI scans, to evaluate the influence of peristalsis.

### 2.4 Statistical analysis

All analyses were performed with the SPSS17.0 and MedCalc statistical software (SPSS 17.0 for Windows, SPSS, Chicago, IL; MedCalc, Mariakerke, Belgium). The intraobserver, interobserver reproducibility and test-retest repeatability of ADC, *D,* and *f* values were analyzed by the paired *t* test. The intraobserver, interobserver reproducibility and test-retest repeatability of *D∗* values were analyzed by the Wilcoxon test, as its distribution was skewed (confirmed by the Kolmogorov-Smirnov test). *P* *<* .05 was considered to indicate statistically significant differences. The repeatability coefficient and Bland-Altman limits of agreements were employed to assess the intraobserver, interobserver reproducibility and test-retest repeatability.^[12,13]^ The repeatability coefficient was defined as 1.96 times the SD of differences between 2 scans or measurements^[13]^ and represented the range of 2 identical measurements for 95% of the subjects. The repeatability coefficient, which represents the threshold value below which the absolute differences between 2 measurements on the same patient are expected to lie for 95% of the measurement pairs, was assessed using the formula 1.96 × dSD (where dSD is the square root of the mean squared difference). For good cohort measurement reproducibility, the repeatability coefficient should be low.

## 3 Results

### 3.1 First scan

The mean values of ADC, *D*, and *f* in rectal cancer were 1.21 ± 0.37 mm^2^/ms, 1.17 ± 0.39 mm^2^/ms, and 13.56 ± 6.74%, respectively. The median value of *D∗* was 14.51 mm^2^/ms (range, 5.00–238.46 mm^2^/ms; percentiles 25th, 5.24 mm^2^/ms; 75th, 50.02 mm^2^/ms; 95th, 228.30 mm^2^/ms).

### 3.2 Second scan

The mean values of ADC, *D*, and *f* in rectal cancer were were 1.18 ± 0.36 mm^2^/ms, 1.13 ± 0.43 mm^2^/ms, 18.61 ± 12.52%, respectively. The median value of *D∗* was 10.65 mm^2^/ms (range, 5.00–85.91 mm^2^/ms; 5th, 5.26 mm^2^/ms; 75th, 21.17 mm^2^/ms; 95th, 84.51 mm^2^/ms).

### 3.3 Intra and interobserver repeatability of the DWI-derived parameters for first scan

There were no significant intra or interobserver differences in the DWI-derived parameters (ADC, *D*, *f*, and *D∗*) measurement for rectal tumor and left gluteal muscle on the first DW-MRI scan (tumor intraobserver: *P* = .973 [ADC], *P* = .256 [*D*], *P* = .088 [*f*], and *P* = .112 [*D∗*]; tumor interobserver: *P* = .098 [ADC], *P* = .454 [*D*], *P* = .381 [*f*], and *P* = .526 [*D∗*]; muscle intraobserver: *P* = .655 [ADC], *P* = .106 [*D*], *P* = .568 [*f*], and *P* = .717 [*D∗*]; muscle interobserver: *P* = .919 [ADC], *P* = .172 [*D*], *P* = .538 [*f*], and *P* = .398 [*D∗*]).

The corresponding repeatability coefficient and Bland-Altman bias are shown in Table 1. The intra and interobserver repeatability coefficient of measurement was higher for rectal cancer than for skeletal muscle, especially the *f* and *D∗* values.

**Table 1:**
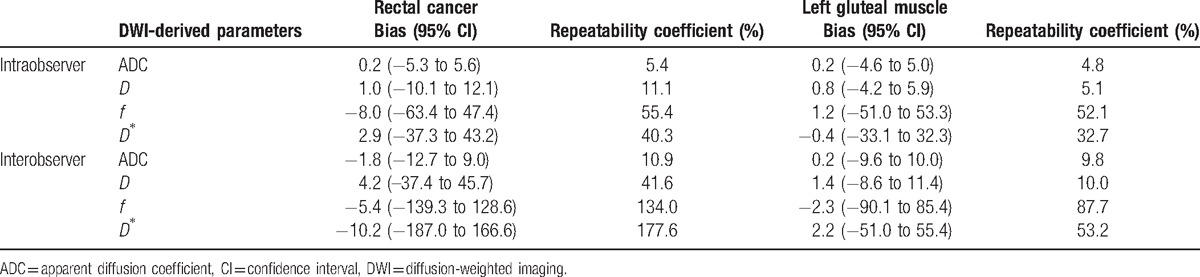
Results of the Bland-Altman repeatability analysis of the DWI-derived parameters.

### 3.4 Test-retest repeatability of the DWI-derived parameters for rectal cancer

**Figure 2:**
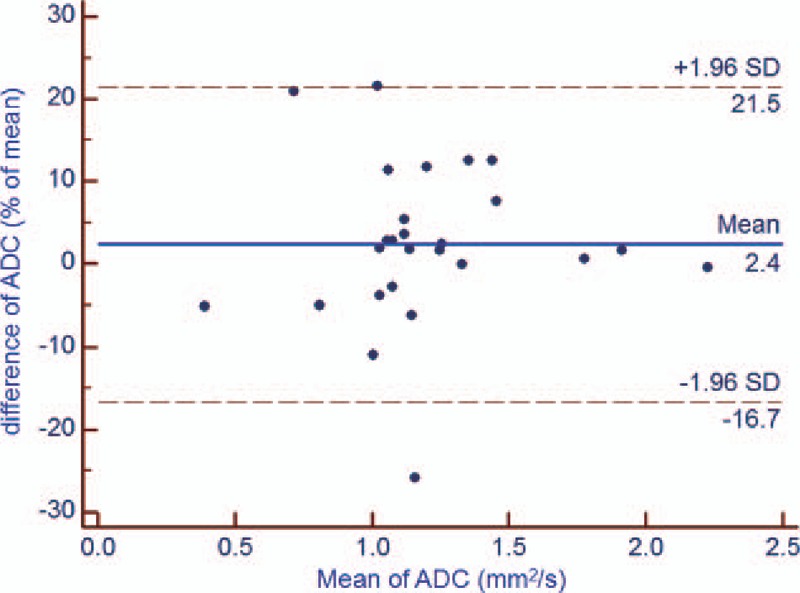
Bland-Altman plot of repeatability of the DWI-derived parameters (ADC) for test and retest DWI imaging of rectal cancer. The horizontal full line represents the bias, and the dotted lines represent the 95% confidence interval. ADC = apparent diffusion coefficient, DWI = diffusion-weighted imaging, SD = standard deviation.

**Figure 3:**
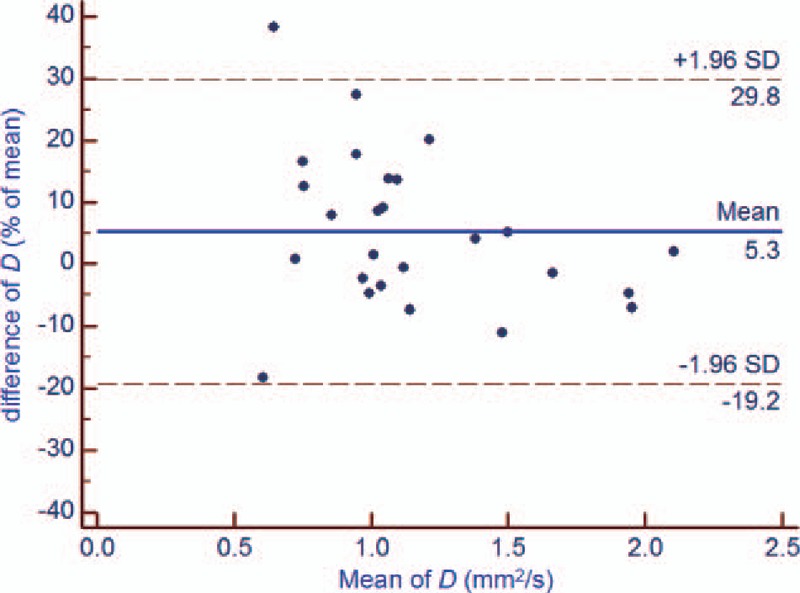
Bland-Altman plot of repeatability of the DWI-derived parameters (*D*) for test and retest DWI imaging of rectal cancer. The horizontal full line represents the bias, and the dotted lines represent the 95% confidence interval. ADC = apparent diffusion coefficient, DWI = diffusion-weighted imaging, SD = standard deviation.

**Figure 4:**
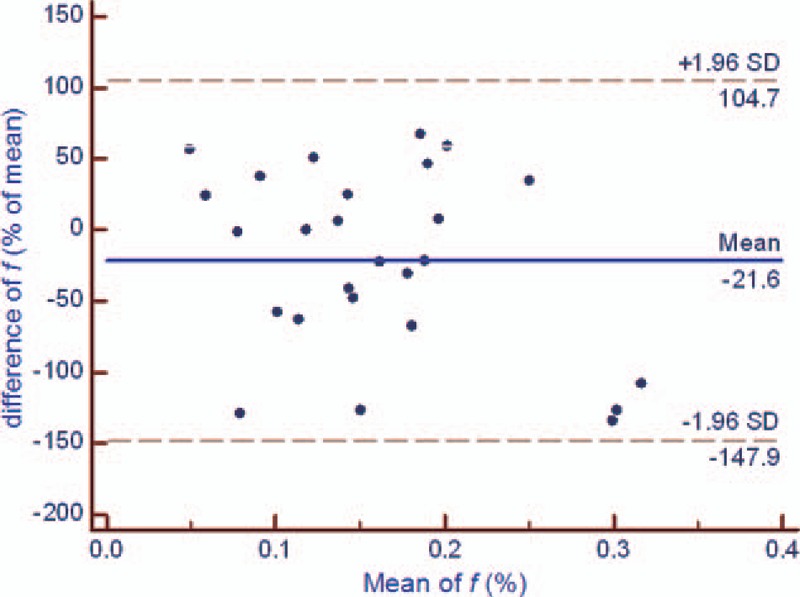
Bland-Altman plot of repeatability of the DWI-derived parameters (*f*) for test and retest DWI imaging of rectal cancer. The horizontal full line represents the bias, and the dotted lines represent the 95% confidence interval. ADC = apparent diffusion coefficient, DWI = diffusion-weighted imaging, SD = standard deviation.

**Figure 5:**
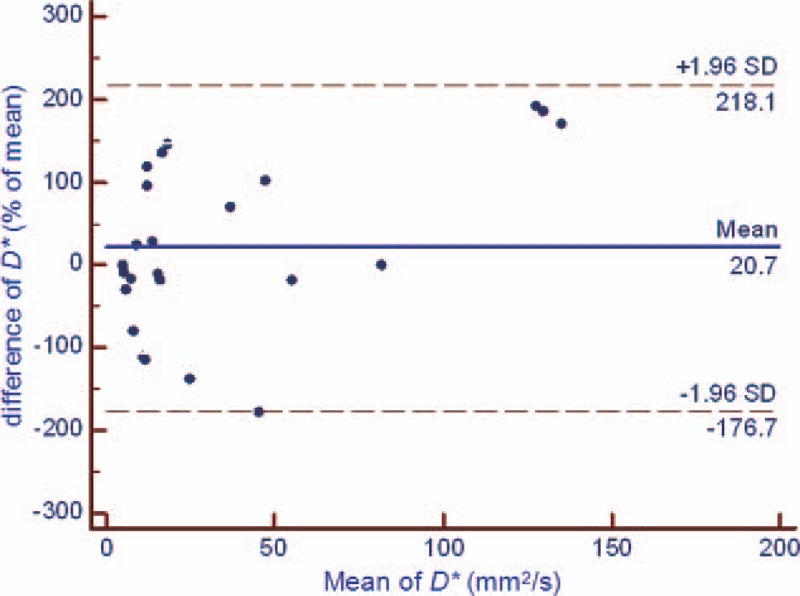
Bland-Altman plot of repeatability of the DWI-derived parameters (*D∗*) for test and retest DWI imaging of rectal cancer. The horizontal full line represents the bias, and the dotted lines represent the 95% confidence interval. ADC = apparent diffusion coefficient, DWI = diffusion-weighted imaging, SD = standard deviation.

There was no significant difference in the test and retest values of the DWI-derived parameters (*P* = .170 [ADC], *P* = .065 [*D*], *P* = .079 [*f*], and *P* = .301[*D∗*]). However, the test-retest repeatability coefficient was higher for IVIM parameters values, especially the *f* and *D*,*∗* than for ADC values in DW-MR imaging of rectal cancer (Table 2, Figs. 2–5). In addition, the test-retest repeatability coefficient was significantly higher than the intraobserver repeatability coefficient.

**Table 2:**
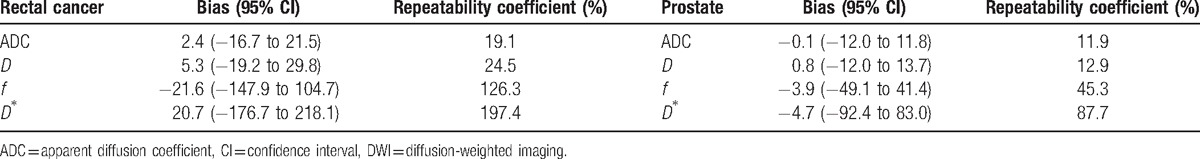
Test-retest repeatability of the DWI-derived parameters for rectal cancer and the prostate.

### 3.5 Test-retest repeatability of the DWI-derived parameters for the prostate

No significant differences were found between the test and retest ADC, *D*, *f*, and *D∗* values of the prostate maximum slice in DWI (*P* = .959 [ADC], *P* = .651 [*D*], *P* = .336 [*f*], and *P* = .624 [*D∗*]). Furthermore, the Bland-Altman bias and repeatability coefficient for all parameters were lower than those for rectal cancer (Table 2).

## 4 Discussion

The intraobserver, interobserver, and test-retest repeatability coefficient was lower for the *D* values than for the *f* and *D∗* values in IVIM-MR imaging of rectal cancer in our study. This result is similar to that of previous studies,^[14,15]^ which reported that the *f* and *D∗* values tended to have greater variability than the *D* values. Furthermore, compared with the results for colorectal metastatic lesions in the liver, the *f* and *D∗* values for rectal cancer showed smaller variability. This is probably related to the extensive neovascular network of rectal cancers; the neovascular network allows for a better signal-noise ratio and more reliable and accurate perfusion-related parameters.^[15]^

In consistent with other studies, results of this study also showed that ADC value, which was calculated by the analytic calculation, had better reproducibility than the IVIM parameters. The main reason of this is that IVIM parameters can only be derived from the nonlinear curve fitting using data from multiple b values. The distribution of b values is thought to be related to the stability of IVIM parameters. However, lack of analytic solution also makes the optimization of b distribution difficult. Lemke et al^[16]^ used Monte-Carlo simulations to optimize the b value distribution. However, Monte-Carlo simulation is too time-consuming and complicated to be employed in the clinical practice. Cohen et al^[17]^ recommended to use more low b values to improve the accuracy of pseudodiffusion parameters. But the study of Dyvorne et al^[18]^ showed that even more b values did not increase the reproducibility of IVIM. So in our study, 8 b values including 5 b values <200 s/mm^2^ were scanned, to balance the accuracy and scan time.

The measurement reproducibility of the DWI-derived parameters is affected by various factors, such as physiological motion, the algorithm used, observer errors, and scanning protocol. Hence, some researchers made an effort to improve the accuracy of the measurement of the DWI-derived parameters. Yedaun et al^[19]^ introduced respiratory and echocardiography-based triggering technology for liver MR examination to reduce physiological motion-induced measurement errors. Freiman et al^[20,21]^ introduced the spatial homogeneity model, and the iterative algorithm used in this model improved the noise robustness of analysis. Because of the presence of air in the rectal cavity, the geometric shape of the rectum would not stay the same during the examination, even when anisodamine hydrochloride is administered before the imaging to inhibit obvious intestine peristalsis. The movement of intrarectal air creates different susceptibility artifacts in DW-MR images, and thus influences the DWI-derived parameters. This is also probably one of the reasons why the test-retest repeatability coefficient was significant higher than the intraobserver repeatability coefficient.^[17]^ Moreover, for male patients in the study, the values of the DWI-derived parameters of the prostate maximum slice were simultaneously derived from 2 identical DW sequences as reference: the repeatability coefficients for the ADC, *D*, *f*, and *D∗* values were lower for the prostate than the rectal cancer. We presume that slight movement of the rectum is a likely contributory factor to the lower measurement reproducibility of the DWI-derived parameters in rectal cancers compared with results obtained from prostate tissue. However, as movement of the rectum is a physiological motion, it cannot be ignored, especially in therapy response assessment; therefore, the best that can be done is to reduce its influence as much as possible.

A previous study showed that increase in ADC values by >40% after therapy could be considered as good response in patients with rectal cancer.^[22]^ Thus, it would be helpful to distinguish the response from measurement errors based on this predicted ADC cutoff value and the relevant repeatability coefficient. IVIM is considered to be more a sophisticated and accurate option to ADC with regard to investigating tissue characteristics with DWI.^[23]^ The theoretical advantages of this approach have not yet been proven in the clinical context. To date, few published studies have documented treatment assessment using IVIM; therefore, further studies are warranted to identify the most robust and accurate assessment parameters. Our study showed that the repeatability coefficient of IVIM parameters has potential in the evaluation of rectal cancer when interpreting positive or negative treatment responses. This study focused on the reproducibility of DWI-derived parameters derived from test-retest DW-MR data obtained in a short time interval; in other words, possible variation between repeat DW-MR scans taken with the same protocol was investigated.

There are several limitations in this study. First, the sample size is relatively small, so there is a possibility of a selection bias. Second, determining the test-retest repeatability of scans with a long interval would represent the actual clinical value better, but in our study, the scan was repeated after an interval of only 20 to 30 minutes based on the patients’ tolerance and clinical work flow. Third, all the data were processed at the same workstation using the same built-in analysis software, and the values were not compared with those obtained with other analysis software. Fourth, we did not include patients with mucinous adenocarcinoma, which has far lower cellular density than that of ordinary tubular adenocarcinomas, consisting of a larger amount of extracelluar mucin and cancer cell columns in the mucinous pool.^[24]^ What's more, signal intensities observed in DW images were quite different between mucinous and ordinary tubular adenocarcinoma.^[25]^ Fifth, we did not perform comparative studies by changing parameters such as the scan interval, the signal-to-noise ratio, reproducing the scan volume and slice orientation and b values, which would affect reproducibility as well. Finally, the spatial correlation among neighboring voxels can be used to improve the noise robustness of IVIM parameter estimations, which could have improved the final repeatability coefficient.^[26]^

In conclusion, good intraobserver reproducibility was observed for the ADC, D, *f*, and D∗ values in DW-MR images. The test-retest reproducibility of ADC and *D* was better than that of *f* and *D∗* for rectal cancer imaging. Therefore, more attention should be given to variance in these parameters, as they reflect the pathophysiological characteristics and treatment response of the rectal cancer. Furthermore, more effort should be invested in improving the reproducibility of DWI-derived parameters so that they are suitable for clinical application.
